# A Method for Real-Time Fault Detection of Liquid Rocket Engine Based on Adaptive Genetic Algorithm Optimizing Back Propagation Neural Network

**DOI:** 10.3390/s21155026

**Published:** 2021-07-24

**Authors:** Huahuang Yu, Tao Wang

**Affiliations:** School of Intelligent Systems Engineering, Sun Yat-sen University, Guangzhou 510006, China; yuhh6@mail2.sysu.edu.cn

**Keywords:** liquid rocket engine, liquid hydrogen and liquid oxygen rocket engine, genetic algorithm, back propagation neural network, fault detection

## Abstract

A real-time fault diagnosis method utilizing an adaptive genetic algorithm to optimize a back propagation (BP) neural network is intended to achieve real-time fault detection of a liquid rocket engine (LRE). In this paper, the authors employ an adaptive genetic algorithm to optimize a BP neural network, produce real-time predictions regarding sensor data, compare the projected value to the actual data collected, and determine whether the engine is malfunctioning using a threshold judgment mechanism. The proposed fault detection method is simulated and verified using data from a certain type of liquid hydrogen and liquid oxygen rocket engine. The experiment results show that this method can effectively diagnose this liquid hydrogen and liquid oxygen rocket engine in real-time. The proposed method has higher system sensitivity and robustness compared with the results obtained from a single BP neural network model and a BP neural network model optimized by a traditional genetic algorithm (GA), and the method has engineering application value.

## 1. Introduction

With the advent of key aerospace programs, such as deep space exploration, crewed spaceflight, lunar exploration, and space station construction, LRE serves as the central power unit and component of the launch vehicle propulsion system. Its reliability and safe operation have become a focus of people’s attention [1]. Effective fault detection and management systems for LRE can help reduce the likelihood of system failure during operation and prevent unwarranted property damage.

Existing fault detection and diagnosis methods for LRE are often classified into three categories: model-driven methods, data-driven methods, and methods based on artificial intelligence [1,2,3]. The model-driven method needs to establish a system model according to the law of system operation. The degree of conformity between the established model and the actual circumstance determines the accuracy of the diagnosis results. However, LRE is a complicated nonlinear system with significant nonlinearity, non-stationarity, and uncertainty, making it challenging to develop an appropriate system model. This method is frequently used in conjunction with others. Ref. [4] performed real-time fault diagnosis on LRE based on the autoregressive moving average (ARMA) model. The data-driven method analyzes the engine’s output signal with respect to the relationship between the system’s output and the fault. This approach requires a large amount of high-quality data to be supported. However, even for the same type of engine, the faults shown may vary due to the engine’s working conditions. This method is more ideal for fault diagnostics and alarming during the engine’s steady condition. Ref. [5] established an adaptive correlation algorithm and envelope method for real-time fault detection and alarm during steady-state and startup processes of LRE. Methods based on artificial intelligence mainly include expert systems, fuzzy theory, neural networks, and others. Ref. [6] developed an artificial neural network-based isolation and replacement algorithm for the fault management of LRE sensors. In the study of LRE fault diagnosis using BP neural network, Ref. [7] proposed a turbopump multi-fault diagnosis method based on a parallel BP neural network. Ref. [8] performed real-time fault diagnosis on LRE based on a dynamic cloud BP network. Ref. [9] proposed an LRE fault detection method based on quantum genetic algorithm optimized BP neural network. Ref. [10] proposed combining the improved particle swarm algorithm and BP neural network. The related research enhanced the convergence speed and defect detection accuracy of BP neural networks, resulting in pretty good results for offline engine fault diagnosis. It is noted that, in Ref. [11], the authors proposed an unsupervised method for diagnosing electric motor faults by utilizing a novelty detection approach based on deep autoencoders and discover that the multi-layer perceptron autoencoder provides the highest overall performance when compared to convolutional neural networks and long short-term memory. The authors of Ref. [12] suggested a technique for state estimation and, consequently, fault diagnostics in nonlinear systems and utilized a simulation of an SLFJR electric drive model to demonstrate their method. To overcome the drawbacks and flaws of fixed detection in cloud computing, Ref. [13] offered an adaptive and dynamic adjustment fault detection model based on support vector machines and decision trees. All of the studies mentioned above have made significant contributions to related subjects.

The study investigates real-time fault detection in LRE and offers a method for real-time fault detection in LRE that utilizes an adaptive genetic algorithm (AGA) to optimize a BP neural network first, followed by a threshold judgment mechanism. The paper compares the suggested model to a single BP neural network and a typical genetic algorithm optimized BP neural network, with a certain type of liquid hydrogen and liquid oxygen rocket engine (LH2/LOX rocket engine) serving as the simulation object. The simulation results demonstrate that the suggested system is capable of real-time fault detection in LRE and that it is also capable of detecting defects more quickly.

The rest of the paper is organized as follows. Section 2 provides an overview of the LH2/LOX rocket engine. Section 3 details the process of developing an AGA optimized BP neural network model. Section 4 discusses how to use the model described in Section 3 for real-time fault detection and introduces the method of threshold detection. Section 5 is a validation of the suggested method using simulation. Section 6 draws conclusions.

## 2. Liquid Hydrogen and Liquid Oxygen Rocket Engine

Rocket engines used in aerospace mainly include solid rocket engines, solid-liquid hybrid rocket engines, conventional propellant engines, liquid oxygen kerosene rocket engines, liquid oxygen methane rocket engines, and LH2/LOX rocket engines. Among these, the LH2/LOX rocket engines powered by liquid hydrogen and liquid oxygen offer unmatched safety, adaptability, reliability, and economics, and have become the focal point of global launch vehicle power system development [14].

**Figure 1:**
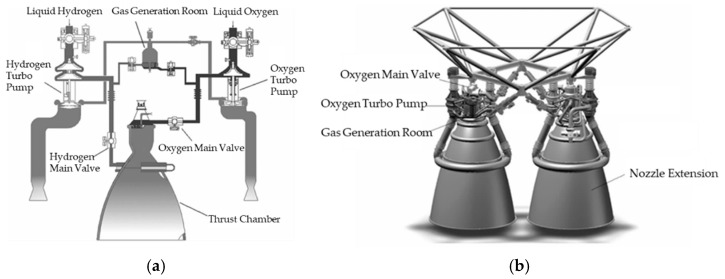
(**a**) The principle of 200-ton LH2/LOX rocket engine; (**b**) the structure of a 200-ton LH2/LOX rocket engine [15].

China, since the 1970s, has successfully developed a 4-ton YF-73 LH2/LOX rocket engine for the CZ-3, an 8-ton YF-75 LH2/LOX rocket engine, and a 9-ton YF-75D and 50-ton YF-77 LH2/LOX rocket engines for CZ-5, and has completed numerous major space launches, including multiple artificial satellites, lunar orbiting probes, lunar exploration projects, and laid the foundation for manned spaceship and space station projects. In the 21st century, when China demonstrated and researched the moon’s landing mode, heavy-duty launch vehicles, and power systems, it determined that the first and booster stages would be powered by a 500-ton liquid oxygen kerosene rocket engine, while the second stage would use a 200-ton LH2/LOX rocket engine. The 200-ton LH2/LOX rocket engine’s operating principle and structure are depicted in Figure 1 [15,16,17,18].

The LH2/LOX rocket engine is mainly composed of a gas generation room, a thrust chamber, a hydrogen main valve, an oxygen main valve, a hydrogen turbo pump, an oxygen turbo pump, pipelines. The sensor data acquired by the system for the purpose of detecting rocket engine faults usually comprise temperature, pressure, speed, pressure, and vibration. [19,20,21,22]. The sensor parameters used in this article include hydrogen pump inlet pressure $P_{ih}$, oxygen pump inlet pressure $P_{io}$, generator pressure before hydrogen injection $P_{gih}$, generator pressure before oxygen injection $P_{gio}$, combustion chamber pressure $P_{c}$, hydrogen pump outlet pressure $P_{eh}$, oxygen pump outlet pressure $P_{eo}$, the chamber pressure $P_{g}$ of the combustion chamber, the temperature $T_{eh}$ after the hydrogen pump, the temperature $P_{eo}$ after the oxygen pump, and the temperature $T_{g}$ of the gas generator.

## 3. Model Building

In the real-time fault detection of LRE, this paper employs an AGA to optimize a BP neural network, and obtains the AGABP model.

### 3.1. BP Neural Network

The BP neural network was proposed in 1986 by a team of scientists led by D.E. Rumelhart and J.L. McClelland. It is a multilayer feedforward network trained by an error back-propagation algorithm. The topology of the BP neural network consists of an input layer, hidden layer, and output layer. As a typical algorithm in artificial intelligence, BP neural networks haveshown that a three-layer BP network can approximate any nonlinear mapping relationship as long as the parameters are appropriate. The BP neural network is widely used in system control, pattern recognition, image recognition, and information processing. However, BP neural networks also have disadvantages, such as slow convergence speed and ease of falling into local extreme points. Using GA to optimize BP neural networks is a way to overcome these drawbacks. This study employs an AGA to optimize the performance of BP neural networks. In the AGABP model, the role of the BP neural network is to perform regression fitting on the acquired sensor parameters.

### 3.2. Adaptive Genetic Algorithm

GA is an adaptive probability optimization technology based on biological genetics and evolutionary mechanisms, created by Professor Holland and his students at the University of Michigan in the United States, and is suitable for optimizing complex systems. GA is an efficient, parallel, and global search method. It can automatically acquire and accumulate information about a search space during the search process and adaptively control all processes to find the optimal solution. In addition, there are many optimization algorithms based on bionics mechanisms for solving different technical applications [23,24,25].

Numerous practices and research [26] have demonstrated that the standard GA has a serious drawback that its crossover and variation probabilities are fixed, implying that both good and bad individuals undergo the same crossover and variation operations. This impairs the algorithm’s local search ability and search efficiency in the late stages of evolution, making it susceptible to premature convergence. In order to overcome the disadvantages of the standard GA, in 1994, Ref. [27] proposed an AGA that dynamically adjusts the crossover probability and mutation probability according to the fitness value. In the early stages of population evolution, a higher crossover probability and variation probability are used to force the population to evolve globally in order to quickly find the optimal solution, while in the later stages, a lower crossover probability and variation probability are used to force the population to evolve locally in order to help the population converge. The adjustment formula used by the AGA is as follows:

(1) $$p_{c}=\{\begin{array}{cc} \frac{k_{1}(f_{\max}-f^{\prime })}{f_{\max}-f_{avg}} & f^{\prime }\geq f_{avg}\\ k_{2} & f^{\prime }<f_{avg} \end{array}$$

(2) $$p_{m}=\{\begin{array}{cc} \frac{k_{3}(f_{\max}-f)}{f_{\max}-f_{avg}} & f\geq f_{avg}\\ k_{4} & f<f_{avg} \end{array}$$

In Formulas (1) and (2), $p_{c}$ represents the probability of crossover, $p_{m}$ represents the probability of mutation, $f_{\max}$ represents the maximum fitness value of the population, $f_{avg}$ represents the average fitness value of the population, $f^{\prime }$ represents the larger fitness value of the two individuals to be crossed, and f represents the mutation value of the fitness value of the individual.

In standard GA, the crossover probability is usually chosen randomly as a larger value and the mutation probability as a smaller value, usually in the range of 0.5–1.0 for the crossover probability and 0.001–0.05 for the mutation probability [28]. To avoid AGA slipping into local optimum solutions, the strategy of individuals with less than average fitness traversing the search space in search of the range containing the ideal solutions is applied. Individuals with poor fitness values should be disrupted as much as possible, while those with high fitness values should be preserved appropriately [29,30]. To accomplish this, in this paper, $k_{1}=0.6$, $k_{2}=0.9$, $k_{3}=0.01$, $k_{4}=0.1$.

The article uses an AGA to optimize the initial weights and thresholds of a BP neural network, and locks the optimal global range in advance through optimization to speed up the BP neural network’s convergence and prevent it from falling into the local optimum.

### 3.3. Using AGA to Optimize BP Neural Network

The process of optimizing BP neural network using AGA may be separated into three stages: determining the structure of the BP neural network; optimizing the BP neural network using AGA; using the optimized BP neural network to forecast.

**Figure 2:**
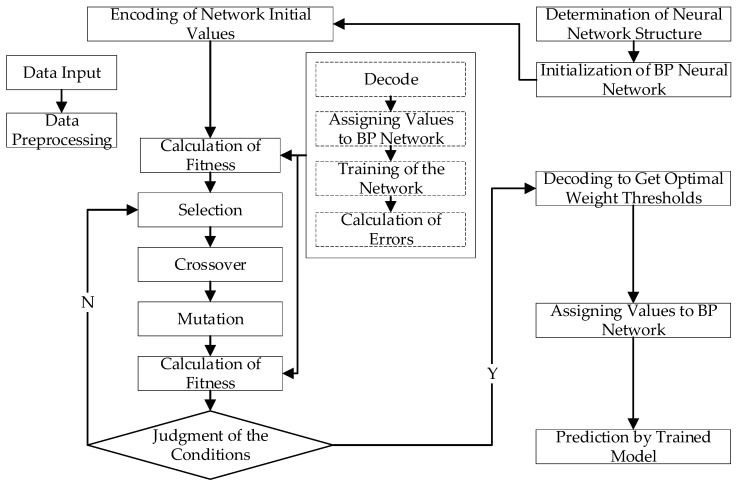
The flow chart for optimizing the BP neural network using AGA.

The flow chart for optimizing the BP neural network using AGA is illustrated in Figure 2.

#### 3.3.1. Determining the Structure of the BP Neural Network

A three-layer structure is chosen for the BP neural network in the article, including an input layer, a hidden layer and an output layer. Using a certain type of LH2/LOX rocket engine as the test object, 11 sensors are selected as data monitoring points. The gas generation chamber pressure $P_{g}$ is selected as the fitting network output, and the remaining sensor data is used as the input to the BP neural network. Therefore, the number of input layer nodes of this network is determined as 10 and the number of output layer nodes is 1.

According to empirical Formula (3), *m* is the number of hidden layer nodes, *n* is the number of input layer nodes, *l* is the number of output layer nodes, and *a* is a constant between 1–10. After repeated simulation verification, the number of hidden layer nodes is determined to be 8, and then the network structure of the model is determined to be 10-8-1.

(3) $$m=\sqrt{n+l}+a$$

The computational complexity calculation method for single BP training of this proposed BP Network is obtained by the following equation [31],

(4) O(b×t×(n×m+m×l))

where *b* is the number of training samples and *t* is the number of iterations. The computational complexity of the above model is O(88 bt) by using Formula (4). It can be seen that after determining the network structure, the computational complexity of this network is related to the number of samples *b* trained and the number of iterations *t*. The richer training samples help to reduce overfitting, but also increase the computational complexity of training. In addition, the size of the number of training iterations *t* has an impact on the computational complexity.

**Figure 3:**
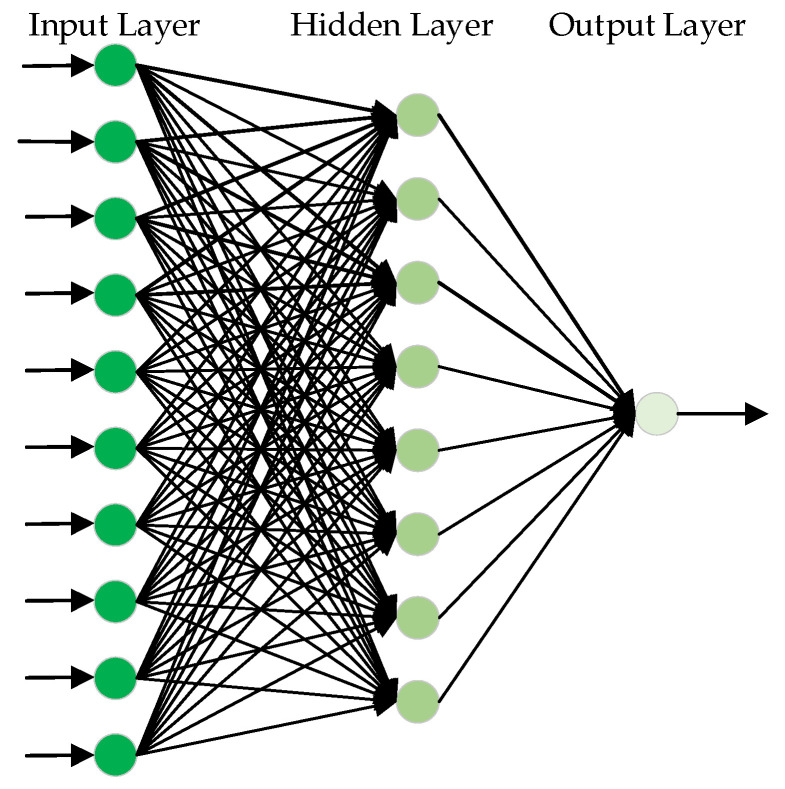
The structure of a BP network (10-8-1).

Figure 3 shows the structure of the BP neural network. The training sample of the network is the data of a certain type of LH2/LOX rocket engine during normal operation.

#### 3.3.2. Optimizing the BP Neural Network Using AGA

Utilizing AGA to optimize the BP neural network’s initial weights and thresholds, allows the network to converge more quickly during future training and avoids the problem of local optimization. The steps to optimize BP using AGA are as follows:

Step 1: Initial population. Use AGA to encode the initial value of the BP neural network to obtain the initial population. The population refers to the effective solution in the problem search space. In the optimization process, a set of effective solutions refers to a BP neural network set of weights and thresholds. The boundary of the neural network weight and the threshold is set to [−1, 1]. As 10-8-1 is the structure of the BP neural network, the length of the chromosome in the AGA is set as 10 × 8 + 8 + 8 × 1 + 1 = 97. The population size determined in this paper is 20. The populations were formed randomly on this basis.

Step 2: Calculate fitness. Calculate the fitness value of each individual in the population. The fitness value is the criterion for judging the quality of this solution. In this article, the reciprocal of the root mean square error of the BP network fitting is used as the fitness value.

Step 3: Selection. Selection is the process of selecting individuals with strong vitality in a group to produce a new group. Individuals with higher fitness values are more likely to produce offspring. In the article, AGA employs a roulette wheel to assign probabilities to individuals and selects them for creating the next generation proportional to their fitness values.

Step 4: Crossover. Crossover refers to the exchange of some genes between two paired chromosomes to form two new individuals. Crossover is the primary method to generate new individuals in GA, and it determines the global search ability of the algorithm. In this paper, the single-point crossover method is used for crossover operation.

Step 5: Mutation. Mutation, the replacement of gene values at some locus in the chromosomal coding strand of an individual with other alleles of that locus, results in the formation of a new individual. Mutation is an auxiliary method to generate new individuals and determines the local search ability of the GA.

Step 6: Calculate the fitness value, check to see if the condition is satisfied, and if not, turn to Step 3 and continue the next round of genetic operation.

Step 7: If the condition is satisfied, the individual with the best fitness value in the output population is used as the optimal solution.

#### 3.3.3. Using the Optimized BP Neural Network to Forecast

After using the AGA to optimize the BP neural network, the optimal weights and thresholds are calculated, the weights and thresholds to AGABP are assigned, and then the resulting model is used to forecast. The specific steps are as follows:

Step 1: Assignment. The values solved by the AGA optimization are assigned to the BP neural network as weights and thresholds.

Step 2: Set training parameters. In this paper, the parameters of the BP neural network are determined as follows: the maximum number of iterations is 1000; the learning rate is 0.01; the training target is 1× 10^−10^. The tansig function is used to define the transfer function between the input and hidden layers, the logsig function is used to define the transfer function between the hidden and output layers, and the negative gradient function is used to create the training function.

Step 3: Train the network to get the expected value.

## 4. Real-Time Fault Detection via AGABP

**Figure 4:**
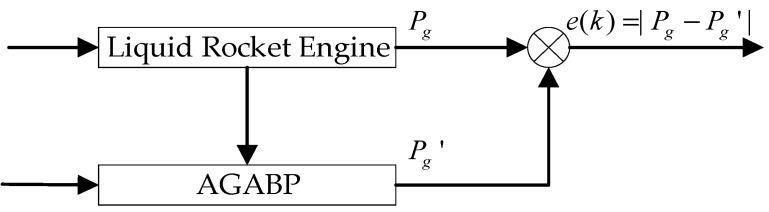
The algorithm diagnosis principle.

The real-time fault detection based on AGABP is achieved by training the model firstly using historical data from the normal engine. Then, the actual measurement data from the engine being tested is used as the input in the model to obtain the real-time predicted value $P_{g}^{\prime }$. Comparing the predicted value $P_{g}^{\prime }$ with the actual sensor measurement value $P_{g}$ the residual is obtained, and the residual is compared with the threshold to determine whether the engine is malfunctioning or not. Figure 4 shows the principle of the AGABP.

**Figure 5:**
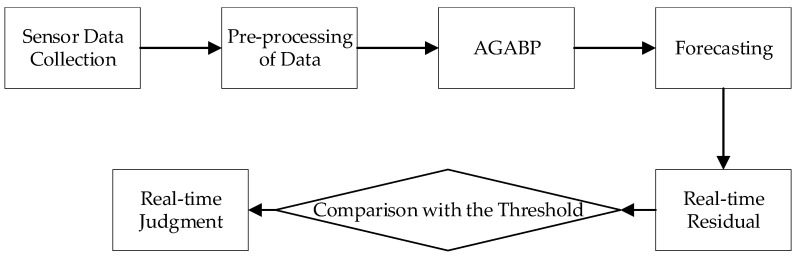
The diagnosis process of AGABP.

Figure 5 shows the diagnosis process of the AGABP model. Specific steps are as follows:

Step 1: Data preprocessing.

Step 2: Use normal engine measured data to train the AGABP model.

Step 3: Process the real-time engine measured data and import it into the established AGABP network model. Then, the model uses the measured values of the other 10 sensors obtained in real-time as input to predict the value of the pressure $P_{g}$ in the gas generating chamber.

Step 4: Compare the predicted value with the currently collected gas generating chamber pressure $P_{g}$ to obtain residual data.

Step 5: Compare the residual error with the set threshold value. When the engine is working normally, the residual error value should be within the threshold value. If the residual error value exceeds the threshold value, that means the engine is malfunctioning.

### 4.1. Data Preprocessing

There are multiple sensors in the LH2/LOX rocket engine that can measure temperature, pressure, speed, vibration, and other types of signals. Different data collectors can be used in the LH2/LOX rocket engine to collect data. When the system is operational, the data acquisition system collects data on the responses of various sensors. These response data have a range of scaling and measurement units that vary. Pre-processing of the collected response data can reduce the interference of other factors on the prediction results.

#### 4.1.1. Select Data

The LH2/LOX rocket engine parameters change continuously with the change in the engine state, especially the change at the moment of ignition. To ensure the correctness of the experimental results, data with obvious mistakes should be deleted prior to training the model, thereby reducing the influence of the error data.

#### 4.1.2. Normalization of Data

The LRE is a complicated nonlinear system that is related to mechanical, flow, and combustion processes, among others. The training samples have a greater influence on the model’s performance, and distinct sensor values have a range and dimension of their own. Prior to diagnosing a liquid rocket engine failure, the engine sensor data must be pre-processed to ensure that all input parameter components are given equal weight at the beginning to solve the numerical effects of the training data on the diagnostic model due to orders of magnitudes. This paper uses the method of normalizing the data for data preprocessing.

The data normalization processing formula is:

(5) $${\bar{x}}_{i}=\frac{x-x_{\min}}{x_{\max}-x_{\min}}$$

Among them, ${\bar{x}}_{i}$ is the normalized data, *x* is the original data, $x_{\min}$ is the minimum value of the data change, and $x_{\max}$ represents the maximum value of the data change. This formula transforms the data into the interval [0,1].

### 4.2. Threshold Judgment Mechanism

When the LH2/LOX rocket engine is working normally, its sensor parameters are stable within a specific dynamic range. When a fault occurs, the sensor’s detection parameters will exceed the normal working range. As a result, the simplest and most effective technique to determine whether a measured signal is normal is to compare the observed value to the permissible limit value (threshold value). This is the oldest and most generally used engineering processing method, as well as one of the most prevalent and widely used methods in spacecraft fault detection systems.

The most critical parameter of threshold-based fault detection is the threshold. Reasonable thresholds have an important impact on determining the time in fault and the stability of the system. Generally, a reasonable threshold is set based on historical experience. When the difference between the two values exceeds the predefined threshold, the system recognizes a problem and issues an early warning. If the threshold is set too high, the system will almost certainly overlook error warnings, but if it is set too low, the system will be overly sensitive and will issue false alarms. In the paper, the threshold setting is determined by the sensor’s average value to be tested in the normal test engine plus a tiny constant. In this method, the threshold is set to 0.2. The measured value is a random quantity. Assuming that *x* is the threshold of the difference between the critical sensors of the LH2/LOX rocket engine, −0.2 is the lower threshold of the normal working range of the sensor, and 0.2 is the upper threshold of the normal working range of the sensor. The normal range of the difference *x* is:

(6) −0.2≤x≤0.2

If only using the threshold criterion, it will quickly lead to misjudgment. To minimize misjudgment and enhance the system’s fault-tolerant alarm capability, the article employs a continuous judgment method; that is, if the residual error between the engine’s predicted and actual values exceeds the threshold five consecutive times, the engine is judged to be faulty; otherwise, it is considered normal.

## 5. Experiment and Simulation Analysis

The following computer platform was used in this article: CPU Intel Core i7-10700, 2.90 GHz eight-core, 64 GB memory. The model was implemented and tested in MATLAB R2020b.

Meanwhile, in order to better observe the simulation results, the simulation in this paper used data of the whole test cycle of a certain type of LH2/LOX rocket engine, mentioned in a previous section. Among them, 730 sets were collected for normal test runs, and 6001 sets of data for faulty test runs were collected. During the test, no interruption simulation processing was performed on the fault. To facilitate comparison of the proposed models’ progress, the methods employing a single BP neural network, an optimized BP neural network using a standard GA, and an optimized BP neural network using an AGA followed by fault detection via a threshold detection mechanism were referred to as the BP model, GABP model, and AGABP model, respectively.

### 5.1. Results about AGABP Model

**Figure 6:**
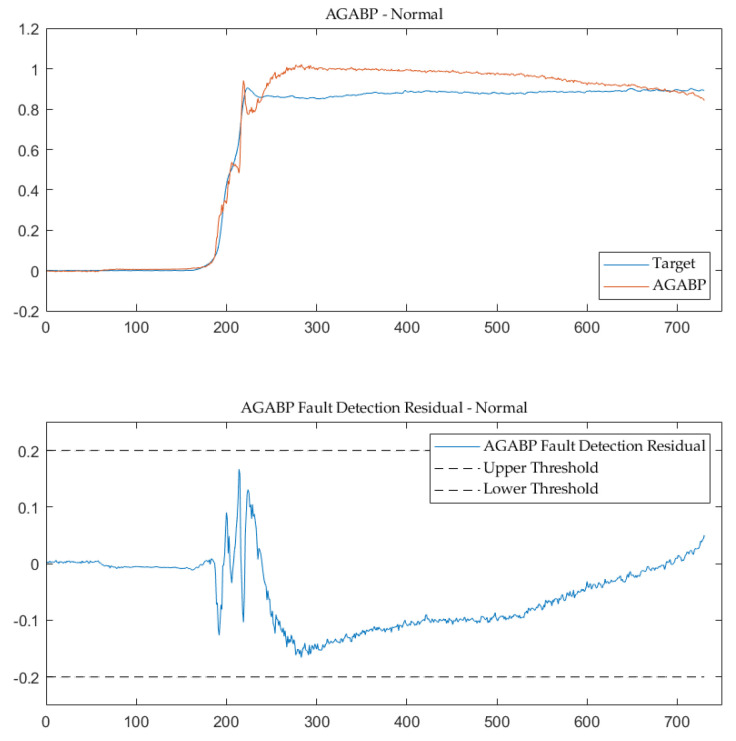
AGABP fault detection—normal.

**Figure 7:**
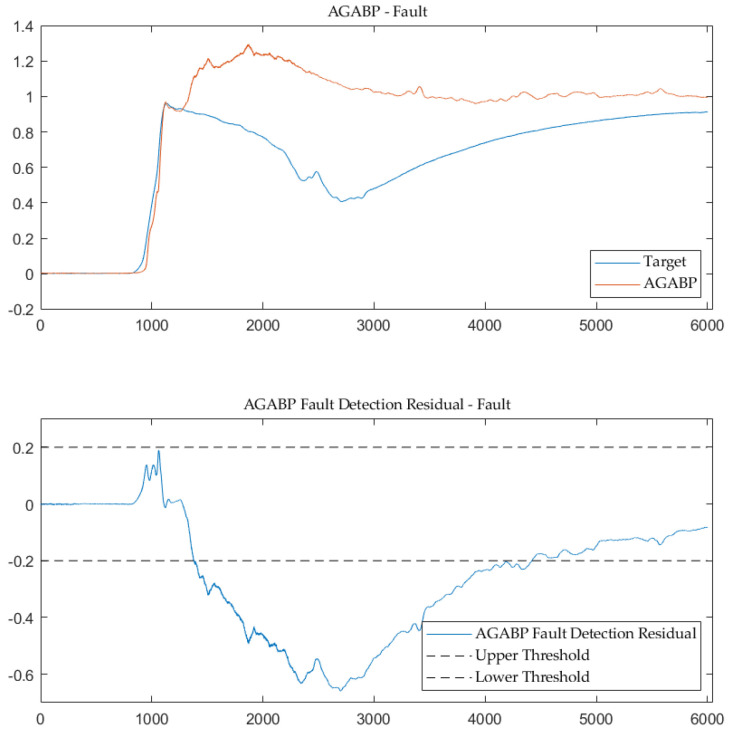
AGABP fault detection—fault.

This paper uses the proposed AGABP model to diagnose the entire process of a certain type of LH2/LOX rocket engine. Figure 6 and Figure 7 are the model’s sensor prediction output of the normal measured data and the measured data at the time of malfunction. As can be seen, the AGABP model provides an accurate diagnosis of the issue, and the 0.2 threshold is reasonable.

### 5.2. Simulation Result and Analysis

In order to evaluate the model proposed in this paper, this paper uses both the BP neural network and the standard GA to optimize the BP neural network to diagnose the faults of the same LH2/LOX rocket engine. In this paper, we ensure that the training parameters of all BP networks are consistent, while for the GABP model, the crossover probability is set to 0.9 and the mutation probability is set to 0.1, which is consistent with the maximum crossover probability and maximum variation probability of the AGABP model. In order to evaluate the quality of each model, the following standards are used as test indicators:

(1) Mean Square Error (MSE). MSE is an estimation function *T* for the unobservable parameter *θ*. Defined as:

(7) $$MSE(T)=E({\left(T-\theta \right)}^{2})$$

(2) Model Forecast Time (MFT). MFT refers to the time when different network models are used to forecast the output results. The time here in this article is the time for predicting a data set for a test cycle.

Table 1 shows the MSE and MFT of each model in processing normal measured data and fault data. The time unit is second.

It can be seen from Table 1 that, among the three detection models tested in this paper, the BP neural network models optimized by genetic algorithm (GABP and AGABP) are far superior to the single BP neural network in terms of computing time. The AGABP model offers a superior detection effect on fault data in MSE evaluation.

**Figure 8:**
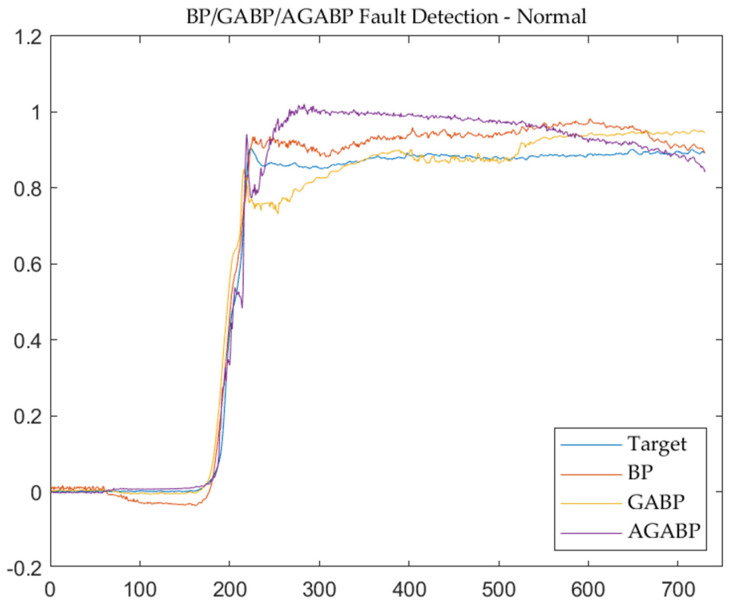
The output of the three models—normal.

**Figure 9:**
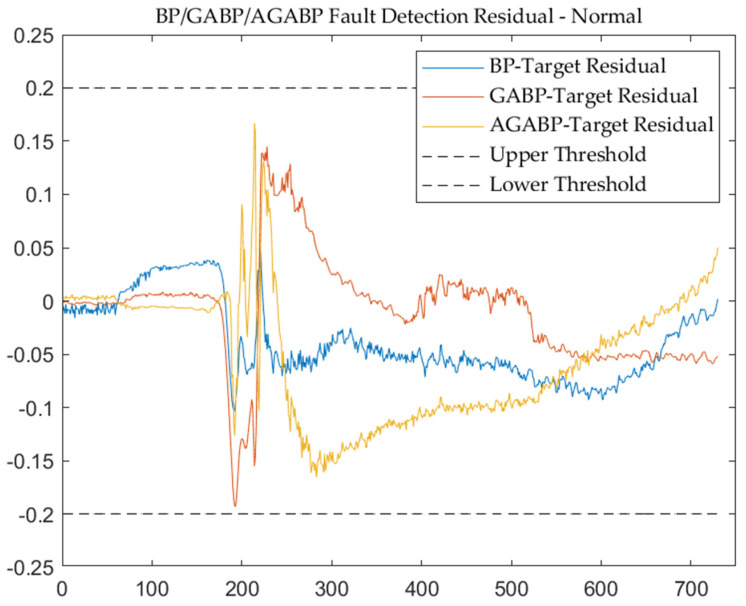
The output residuals of the three models—normal.

Figure 8 and Figure 9 are the output and residual diagrams of the measured data from the three models. It can be seen that each model can correctly judge that the engine is in a normal working state under the condition of a threshold of 0.2.

Comparing the results of the three models shows that in the judgment of normal data, the overall performance of the BP model is more stable. In contrast, the performance of the GABP and AGABP models is relatively volatile. When the engine is ignited (the sample point in the picture is around 200), the changes in each model are drastic, and it is also quite easy to make an error at this time.

**Figure 10:**
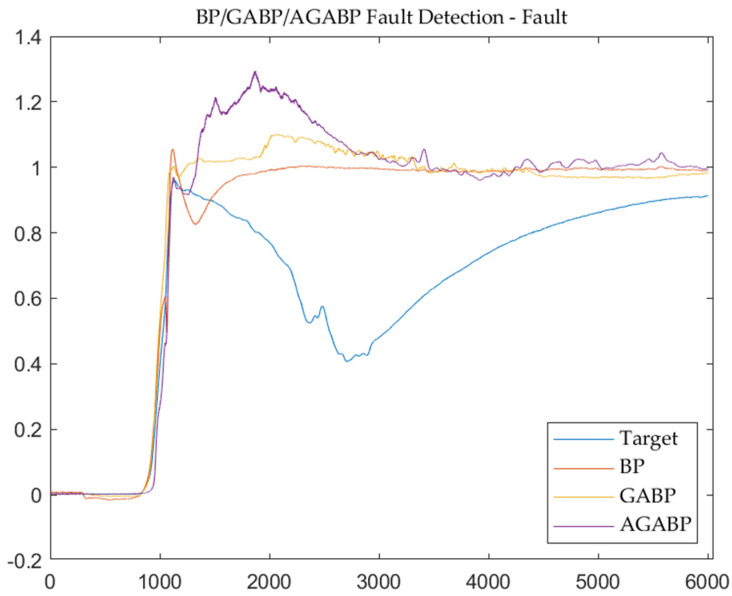
The output of the three models—fault.

**Figure 11:**
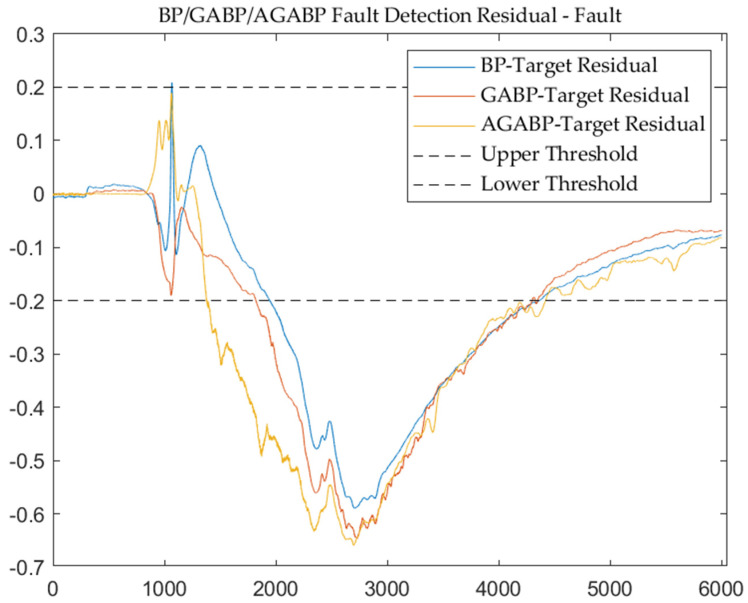
The output residual of the three models—fault.

Figure 10 and Figure 11 are the output and residual diagrams of the three models when diagnosing fault data. Under the condition that the threshold value of each model is 0.2, the three models can correctly judge that the engine is in a fault state. In the data analysis of the failure test, it can be seen that AGABP can diagnose the failure more effectively, and as illustrated in Figure 11, the AGABP model has a faster diagnosis time, a broader range of change, and a more vivid display of failure information.

## 6. Conclusions

The article studies a real-time fault detection method of LRE based on an adaptive genetic algorithm optimized BP neural network, and simulates and analyzes a certain type of LH2/LOX rocket engine as an example. The following conclusions can be obtained:

(1) By using a BP neural network to forecast sensor data and then determining whether a fault occurs via a threshold detection mechanism, the technology can serve as an effective early warning system for engine failure.

(2) In the detection process, the real-time fault detection method based on AGA optimized BP neural network meets the real-time requirements and has engineering application value.

(3) The AGABP model can accurately, stably, and timely output the predicted value of the physical quantity to be measured. It is not easy to fall into the optimal local solution. Using the AGABP model to detect the measured data of a certain type of LH2/LOX rocket engine, the detection results are closer to the actual situation and faster response than those of the BP model, indicating that the method can be well applied to the fault detection of this type of LH2/LOX rocket engine.

(4) Compared with the BP and GABP models, the AGABP model is more suitable for detecting this type of LH2/LOX rocket engine.

## Figures and Tables

**Table 1:** The MSE and MFT of each model in normal data and fault data.

Model	Normal Data (730 Sets)		Fault Data (6001 Sets)	
	MSE	MFT (s) ^1^	MSE	MFT (s) ^2^
BP	0.0027	0.0231	0.0703	0.0665
GABP	0.0026	0.0067	0.0597	0.0073
AGABP	0.0062	0.0054	0.1053	0.0071

^1^ The MFT here refers to the time used to predict 730 sets of data. ^2^ The MFT here refers to the time used to predict 6001 sets of data.

## Data Availability

The data is not publicly available as it involves sensitive information.

## Abbreviations

The following abbreviations are used in this manuscript:

AGA	Adaptive genetic algorithm
AGABP	Adaptive genetic algorithm optimized BP neural network
ARMA	Autoregressive moving average
BP	Back propagation
GA	Genetic algorithm
GABP	Standard genetic algorithm optimized BP neural network
LH2/LOX	Liquid hydrogen and liquid oxygen
LRE	Liquid rocket engine
MFT	Model Forecast Time
MSE	Mean Square Error
